# Automated detection of pain levels using deep feature extraction from shutter blinds-based dynamic-sized horizontal patches with facial images

**DOI:** 10.1038/s41598-022-21380-4

**Published:** 2022-10-14

**Authors:** Prabal Datta Barua, Nursena Baygin, Sengul Dogan, Mehmet Baygin, N. Arunkumar, Hamido Fujita, Turker Tuncer, Ru-San Tan, Elizabeth Palmer, Muhammad Mokhzaini Bin Azizan, Nahrizul Adib Kadri, U. Rajendra Acharya

**Affiliations:** 1 School of Business (Information System), University of Southern Queensland, Toowoomba, QLD 4350 Australia; 2 Faculty of Engineering and Information Technology, University of Technology Sydney, Sydney, NSW 2007 Australia; 3 Department of Computer Engineering, College of Engineering, Kafkas University, Kars, Turkey; 4 Department of Digital Forensics Engineering, College of Technology, Firat University, Elazig, Turkey; 5 Department of Computer Engineering, College of Engineering, Ardahan University, Ardahan, Turkey; 6 Rathinam College of Engineering, Coimbatore, India; 7 Faculty of Information Technology, HUTECH University of Technology, Ho Chi Minh City, Viet Nam; 8 Andalusian Research Institute in Data Science and Computational Intelligence, University of Granada, Granada, Spain; 9 Regional Research Center, Iwate Prefectural University, Iwate, Japan; 10 Department of Cardiology, National Heart Centre Singapore, Singapore, Singapore; 11 Duke-NUS Medical School, Singapore, Singapore; 12 Centre of Clinical Genetics, Sydney Children’s Hospitals Network, Randwick, 2031 Australia; 13 School of Women’s and Children’s Health, University of New South Wales, Randwick, 2031 Australia; 14 Department of Electrical and Electronic Engineering, Faculty of Engineering and Built Environment, Universiti Sains Islam Malaysia (USIM), Nilai, Malaysia; 15 Department of Biomedical Engineering, Faculty of Engineering, University Malaya, 50603 Kuala Lumpur, Malaysia; 16 Department of Electronics and Computer Engineering, Ngee Ann Polytechnic, Singapore, 599489 Singapore; 17 Department of Biomedical Engineering, School of Science and Technology, SUSS University, Singapore, Singapore; 18 Department of Biomedical Informatics and Medical Engineering, Asia University, Taichung, Taiwan

**Keywords:** Health care, Medical research

## Abstract

Pain intensity classification using facial images is a challenging problem in computer vision research. This work proposed a patch and transfer learning-based model to classify various pain intensities using facial images. The input facial images were segmented into dynamic-sized horizontal patches or “shutter blinds”. A lightweight deep network DarkNet19 pre-trained on ImageNet1K was used to generate deep features from the shutter blinds and the undivided resized segmented input facial image. The most discriminative features were selected from these deep features using iterative neighborhood component analysis, which were then fed to a standard shallow fine k-nearest neighbor classifier for classification using tenfold cross-validation. The proposed shutter blinds-based model was trained and tested on datasets derived from two public databases—University of Northern British Columbia-McMaster Shoulder Pain Expression Archive Database and Denver Intensity of Spontaneous Facial Action Database—which both comprised four pain intensity classes that had been labeled by human experts using validated facial action coding system methodology. Our shutter blinds-based classification model attained more than 95% overall accuracy rates on both datasets. The excellent performance suggests that the automated pain intensity classification model can be deployed to assist doctors in the non-verbal detection of pain using facial images in various situations (e.g., non-communicative patients or during surgery). This system can facilitate timely detection and management of pain.

## Introduction

Pain is a sensation emanating from a specific part of the body that can induce protective brain-mediated reflex actions to avert further pain and other automatic responses, such as facial expressions^1,2^. The individual’s pain response differs from person to person and is influenced by subjective pain thresholds, past experiences, and emotional factors^3^. There are two types of pain: acute and chronic^4^. Acute pain is short-term pain that starts suddenly in critical illness or after injury. In contrast, chronic pain occurs over a long time (felt for days to weeks, or even longer), with a long term negative impact on patients' quality of life^5^. Acute and chronic pain symptoms can overlap, for example, acute pain can transition into chronic pain. In the clinic, doctors mostly rely on self-reporting by patients to determine the presence and intensity of pain^6^. Evaluation tools such as numerical rating and visual analog scales have been used in self-reporting of pain^7^ and are highly dependent on effective patient-doctor communication. Not unexpectedly, factors that limit patients’ ability to express themselves, including extremes of age, cultural barriers, and speech disorders or cognitive impairments, can render this process challenging^8^.

Increasingly, researchers are focusing on automatic non-verbal pain detection systems using machine learning models^1,9^. For instance, pain is reflected in the human face, and patients’ facial expressions can provide essential clues to the presence of pain and its severity^10^. In the literature, different automated techniques have been proposed using facial images for pain detection. Very young age and limited language skills preclude pain self-reporting, which raises the clinical need for automated pain detection systems. Brahnam et al.^11^ proposed a pain detection method for neonates. Using a support vector machine classifier, they studied 204 facial images of 26 neonates and attained 88.00% classification accuracy. In a subsequent publication, Brahnam et al. reported a 100.0% classification rate in their study of 204 facial images of 13 male and 13 female Caucasian neonates^12^; and 90.20% accuracy rate for neonatal pain detection on facial images using a model based on simultaneous optimization algorithm classifier^13^. The experimental protocols differed in^12^ and^13^. In^12^, all subjects’ facial images were included in both training and testing sets, whereas in^13^, the testing set contained a separate set of unseen facial images of subjects. Kristian et al.^14^ proposed an infant pain classification model based on a local binary pattern for feature extraction, which was trained and tested on a dataset^15^ comprising 132 facial images of three classes (“severe pain”, “light to moderate pain”, and “no pain”) that had been regrouped into two classes (“cry” vs. “no cry”). They reported 92.30% classification accuracy. Studies of pain detection using facial images among adults generally garnered less impressive performance, plausibly due to adult facial expressions' wider variety and dynamic range. Othman et al.^16^ used MobilNetV2 to detect pain detection on facial images from two databases: BioVid Heat Pain^17^ and X-ITE Pain^18^ databases, which collected videos of facial expressions as well as other biopotential signals from 87 (8700 samples) and 134 subjects, respectively. They reported 65.5%, 71.4%, and 72.6% accuracy rates on the BioVid, X-ITE, and combined BioVid + X-ITE databases, respectively. Weitz et al.^19^ applied deep learning for pain detection using a dataset comprising facial images belonging to three classes: “pain” (4692 images), “disgust” (4815 images), and “happiness” (4815 images). They reported precision, recall, and F1-score of 67.00%, 66.00%, and 66.00%, respectively. Yang et al.^20^ proposed an automated pain assessment model using facial videos based on local binary patterns, binarized statistical image features, and local phase quantization. Two datasets—comprising 129 subjects with shoulder pain^21^ and 90 subjects receiving painful heat stimuli (BioVid)^17^—were used to develop the model. They reported accuracy rates of 83.42% and 71.00% for the shoulder pain and BioVid datasets, respectively. Kharghanian et al.^22^ developed a convolutional deep belief network model for pain detection using facial image data belonging to 25 subjects with shoulder pain. They reported accuracy, F1-score, and area under the receiver operating characteristic curve of 87.20%, 86.44%, and 94.48%, respectively. Zafar and Khan^23^ proposed an automated system for pain intensity classification using a shoulder pain dataset^21^ comprising 200 videos (14,670 training and 6830 testing video frames), and attained 84.02% classification accuracy. A summary of previously published studies on automated pain levels detection using facial images is provided in Table 1.

**Table 1:** Summary of the state-of-the-art for automated pain level detection using facial images.

Study	Method	Classifier	Number of subjects	Number of facial images/frames	Accuracy (%)	Limitations
Brahnam et al.^11^	Principal component analysis, linear discriminant analysis	Support vector machine	26 (13 male, 13 female)	204	88.00	Single and small dataset, low accuracy
Brahnam et al.^12^	Principal component analysis, linear discriminant analysis, frequency domain methods	Neural network simultaneous optimization algorithm	26 (13 male, 13 female)	204	100.0	Single and small dataset
Brahnam et al.^13^	Principal component analysis, linear discriminant analysis	Neural network simultaneous optimization algorithm	26 (13 male, 13 female)	204	90.20	Single and small dataset
Kristian et al.^14^	Active shape model, local binary pattern	Support vector machine	23	132	88.70	Single and small dataset
Othman et al.^16^	MobileNetV2	Softmax	1. 87	1. 3480	1. 6550	Low accuracy
			2. 134	2. 7763	2. 7140	
Weitz et al.^19^	Convolutional neural network	Softmax	324	14,322	67.00	Single dataset, low accuracy
Yang et al.^20^	Local binary pattern, local phase quantization, statistical features	Support vector machine	1. 129	1. 48,398	1. 8342	Low accuracy
			2. 90	2. 8700	2. 71.00	
Kharghanian et al.^22^	Convolutional deep belief network model	Support vector machine	25	48,398	87.20	Single dataset, low accuracy
Zafar and Khan^23^	Geometric features	k-nearest neighbor	Unspecified	21,500	84.02	Single dataset, low accuracy

Facial images contain hidden information and have been commonly used to develop machine learning models, including automated pain intensity detection, a burgeoning area of research. We aimed to develop an accurate pain intensity detection model with acceptable computational time complexity. We were inspired by patch-based feature engineering^24,25^, which has been shown to deliver excellent classification results and transfer learning using pre-trained networks, which can reduce model time complexity without compromising performance^26–28^. To this end, a novel deep feature generator model was proposed. The input image was divided into dynamic-sized horizontal patches—“shutter blinds”—for deep feature extraction using a pre-trained network. The shutter blinds-based feature extraction function was coupled to an efficient downstream feature selection function and shallow classifier to create the final model.

Major contributions of our shutter blinds-based model are:

- Inspired by the ability of spontaneous facial expressions to reflect pain intensity^29^ and patch-based learning models^24,25^, we proposed a novel shutter blinds-based model for extracting deep features from facial images for pain intensity classification. The model exploits the superior performance of deep^30,31^ and patch-based models^30,31^ to extract hidden signatures from facial images using “shutter blinds”, i.e., horizontal dynamic-sized patches.
- Many computer vision applications have demonstrated high classification performance using transfer learning. Transfer learning using pre-trained deep networks was employed in this model to extract deep features with low time complexity^32^.
- The shutter blinds-based classification model attained over 95% classification accuracy rates for pain intensity detection on facial images derived from two established facial image databases.

## Results

The facial images were downloaded from two public datasets, and the model was trained and tested on these two separate datasets in parallel.

The experiments were implemented in MATLAB 2021a programming environment using a laptop with Intel i7 @ 4.7 GHz processor, 32 GB main memory, and 256 GB external hard disk memory, without using any graphics processor. The used datasets were downloaded from the web. The facial images of these datasets were labeled using Eq. (1), and four categories were created. The pre-trained DarkNet19 was imported into MATLAB 2021a as a transfer learning model. Vision cascade object detection tool was used to segment the faces on the input video frames. In this work, we used only the facial area of the image. Feature extraction and selection phases were coded using m files. MATLAB 2021b classification learner tool was used in the classification phase to calculate classification results using various available classifiers. The best-forming fine kNN classifier was chosen for the final model. The presented model is a parametric image classification model and the parameters used are given below.

*Feature extraction function:* Pre-trained DarkNet19. Using the last pooling layer, 1,000 features were extracted using this function.

*Number of used horizontal patches:* 21.

*Number of features:* 21 × 1000 (extracted from patches) + 1000 (extracted from original facial image) = 22,000.

*INCA:* Iteration range was 100–1000 (i.e., 901 features were selected). The loss/error function was kNN with tenfold cross-validation.

*Classifier:* kNN (k was 1; the distance was Manhattan) with tenfold cross-validation.

The presented shutter blinds-based deep feature engineering architecture is versatile and expandable. It can be coupled to other methods, or individual parameter settings of the components can be tweaked to optimize the architecture for solving other image classification problems.

One of the commonly preferred validation methods is tenfold cross-validation. Using tenfold CV, the images were divided into ten folds randomly, one-fold was utilized as test data, and the other nine folds were used to train (similar to 90:10 hold out validation). This operation was repeated ten times, and the calculated average performance measurements were given.

Standard classification performance metrics, namely F1-score, precision, recall, accuracy, Cohen’s kappa, Matthew’s correlation coefficient^33^, and confusion matrices (category-wise recall, precision, and F1-score), were used to evaluate the model.

Class-wise and overall (average of calculated metrics) results are presented separately from the UNBC-McMaster and DISFA datasets. The best recall results were seen in the PSPI > 3 class, with 96.23% class-wise recall (Table 2) for the UNBC-McMaster dataset. The overall accuracy, unweighted average recall, unweighted average precision, average F1 score, Matthew’s correlation coefficient, Cohen’s kappa, and geometric mean were 95.57%, 95.59%, 95.79%, 95.67%, 94.14%, 93.93%, and 95.58%, respectively. The best recall results were also seen in the PSPI > 3 class, with 98.62% class-wise recall (Table 3) for the DISFA dataset. The overall accuracy, unweighted average recall, unweighted average precision, average F1 score, Matthew’s correlation coefficient, Cohen’s kappa, and geometric mean were 96.06%, 96.04%, 96.16%, 96.08%, 94.78%, 94.74%, and 96.03%, respectively.

**Table 2:** Confusion matrix obtained for proposed shutter blinds model using UNBC-McMaster Shoulder Pain Expression Archive Database with tenfold cross-validation.

Real outputs	Predicted outputs			
	PSPI = 0	PSPI = 1	2 ≤ PSPI ≤ 3	PSPI > 3
PSPI = 0	2342	67	59	15
PSPI = 1	11	2798	99	1
2 ≤ PSPI ≤ 3	9	94	3598	62
PSPI > 3	8	4	52	1633
Recall (%)	94.32	96.18	95.62	96.23
Precision (%)	98.82	94.43	94.49	95.44
F1-score (%)	96.52	95.30	95.05	95.83

**Table 3:** Confusion matrix obtained for proposed shutter blinds model using DISFA Database with tenfold cross-validation.

Real outputs	Predicted outputs			
	PSPI = 0	PSPI = 1	2 ≤ PSPI ≤ 3	PSPI > 3
PSPI = 0	8528	405	80	12
PSPI = 1	132	9606	227	8
2 ≤ PSPI ≤ 3	19	338	9765	187
PSPI > 3	4	11	121	9739
Recall (%)	94.49	96.32	94.72	98.62
Precision (%)	98.21	92.72	95.80	97.92
F1-score (%)	96.32	94.49	95.26	98.27

**Figure 1:**
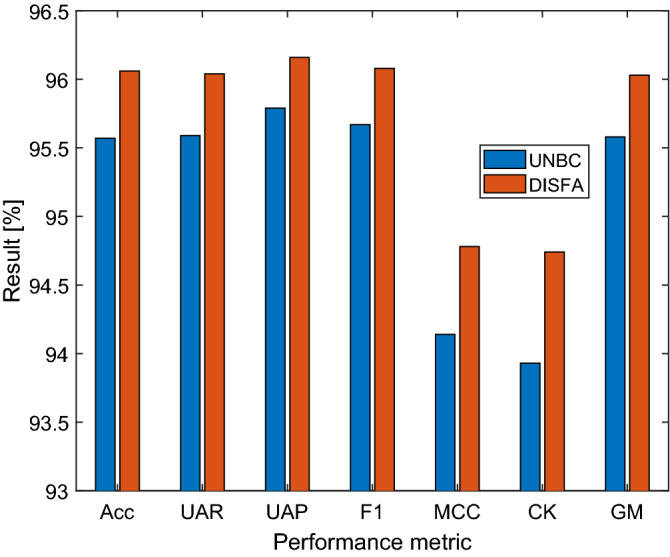
Overall performance comparisons. Acc: accuracy, UAR: unweighted average recall, UAP: unweighted average precision, F1: F1-score, MCC: Matthew’s correlation coefficient, CK: Cohen’s kappa, GM: geometric mean.

The overall results of the UNBC and DISFA datasets are compared in Fig. 1, which demonstrates that the results obtained on the DISFA dataset were about 0.5% higher across all metric compared with the UNBC dataset.

## Discussion

**Figure 2:**
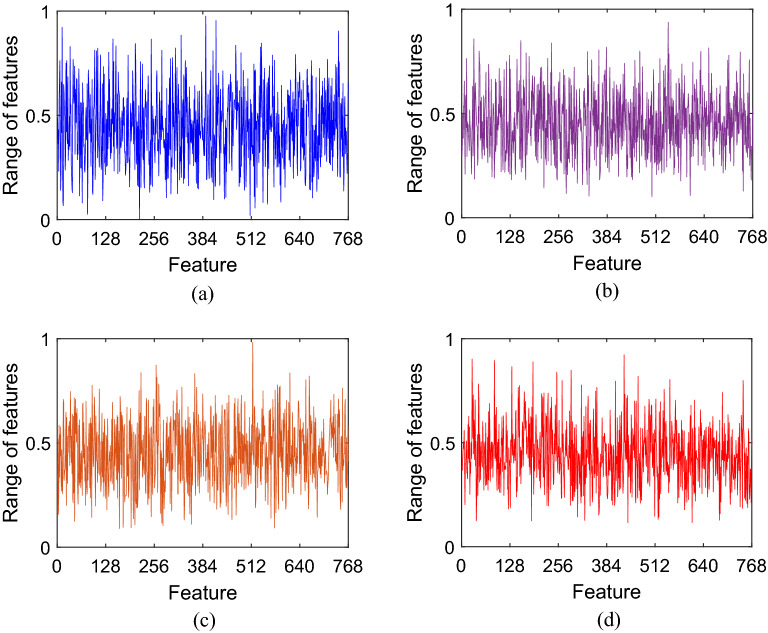
Generated feature vector samples for the different classes of the UNBC-McMaster dataset: (**a**) PSPI = 0, (**b**) PSPI = 1, (**c**) 2 ≤ PSPI ≤ 3, (**d**) PSPI > 3.

This work introduced a new deep learning-based pain intensity classification model. The key feature engineering step involved dividing input video frame images into dynamic-sized horizontal patches—“shutter blinds”—for downstream deep feature extraction using pre-trained DarkNet19^34^. The input video frame images of facial expressions derived from two well-known facial recognition public image databases had been encoded by certified coders using validated FACS methodology and scored into ordinal PSPI categories of pain intensities. The novel shutter blinds-based feature extractor generated 22,000 features from each input image. INCA was used to select 768 and 834 features from the UNBC-McMaster and DISFA datasets, respectively. Using a fine kNN classifier with a tenfold CV strategy for automated classification, our proposed shutter blinds-based model attained excellent performance with overall accuracy rates of 95.57% and 96.06% for the UNBC-McMaster and DISFA datasets, respectively. Of note, the DISFA database is typically used for general facial expression recognition experiments but has been adapted for pain intensity ascertainment in this study. These results affirm the discriminative classification utility of the presented shutter blinds-based model for automated pain intensity classification. In addition, the confusion matrices (Tables 2 and 3) indicate low misclassification rates. As can be seen in Fig. 2, the features generated by our proposed model were distinctive, which help explain the above 95% classification accuracy rates that we have attained for both facial image datasets.

**Figure 3:**
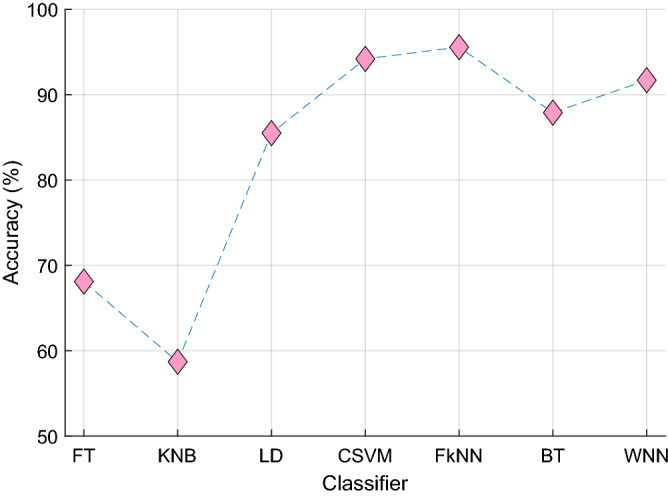
The classification accuracy of seven standard shallow classifiers was attained using tenfold cross-validation on the UNBC-McMaster dataset. The FT, fine tree; KNB, kernel naïve Bayes; LD, linear discriminant; CSVM, cubic support vector machine; FkNN, fine k-nearest neighbor; BT, bagged tree; WNN, wide neural network.

In our final model, we chose pre-trained DarkNet19 to extract features from the “shutter blind” patches and the undivided resized segmented input facial images as it had outperformed other competitors' pre-trained deep models. On the UNBC-McMaster dataset, the classification accuracy rates for DarkNet19, DarkNet53, MobileNetV2, and ResNet50 were 95.57%, 95.02%, 95.41%, and 95.20%, respectively, with a fine kNN classifier using tenfold cross-validation. INCA selected 768, 989, 968 and 970 features from the 22,000 DarkNet19-, DarkNet53-, MobileNetV2-, and ResNet50-generated features per image, respectively, using the UNBC-McMaster dataset. This indicates that DarkNet19’s superior classification accuracy had been achieved efficiently using less selected features (and with less downstream computational cost). We also compared various standard shallow classifiers in the MATLAB2021a classification learner tool in this work. Among fine tree, kernel naïve Bayes, linear discriminant, cubic support vector machine, fine kNN, bagged tree, and wide neural network classifiers, fine kNN attained the best performance (Fig. 3).

We performed a nonsystematic review of the literature on state-of-the-art techniques for pain classification using facial images. The results are summarized in Table 4. All the studies used deep models. Our proposed model attained the highest classification performance. Of note, while all studies in Table 4 have used the UNBC-McMaster database, our study is the only one that uses the DISFA dataset. Although the DISFA database has not been designed primarily for pain classification, AUs attributable to pain response and PSPI scores across a broad range can be identified and scored, respectively, by certified experts in substantial proportions of subjects at rates commensurate with those observed in the UNBC-McMaster Shoulder Pain Expression Archive Database (Table 5), which has been designed specifically for the investigation of pain intensity. Furthermore, the results attained by our model on the DISFA dataset are excellent (Table 2) and comparable with those obtained on the UNBC-McMaster dataset (Table 3). To the best of our knowledge, the current study is the first to adopt the DISFA database successfully for pain intensity classification using validated FACS methodology to characterize AUs implicated in pain response in individual video frames of facial expressions.

**Table 4:** Comparison of our work with state-of-the-art methods developed for automated pain intensity classification using facial images.

Study	Method	Classifier	Dataset	Results
Bargshady^35^	Temporal convolutional network, LSTM, principal component analysis	Temporal convolutional network	UNBC-McMaster (10,783 frames)	MSE: 1.186
				MAE: 0.234
				Acc: 94.14%
				AUC: 91.30%
Bargshady^36^	Ensemble neural network	Ensemble CNN-recurrent neural network	UNBC-McMaster (10,783 frames)	AUC: 90.50%
				Acc: 86.00%
				MSE: 0.081
Semwal^37^	Ensemble of compact CNN	Ensemble	UNBC-McMaster (16,000 frames)	Pre: 91.97%
				Rec: 91.01$
				F1: 91.42%
				Acc: 93.87
Rudovic^38^	CNN	Softmax	UNBC-McMaster (48,106 frames)	Acc: 76.00%
				PR-AUC: 59.00
				F1: 47.00
Karamitsos^39^	CNN	Softmax	UNBC-McMaster (48,398 frames)	Acc: 92.50%
Semwal^40^	CNN	Softmax	UNBC-McMaster (16,000 frames)	Acc: 92.00%
				MAE: 0.20
				MSE: 0.17
Bargshady^1^	CNN, bidirectional LSTM	Enhanced joint hybrid-CNN-bidirectional LSTM	UNBC-McMaster (10,783 frames)	Acc: 91.20%
				AUC: 98.40%
El Morabit and Rivenq^41^	Vision Transformer, Feed Forward Network	Softmax	UNBC-McMaster (48,398 frames)	Acc: 84.15
Our model	Transfer learning, novel shutter blinds-based deep feature extraction	kNN	UNBC-McMaster (10,852 frames)	Acc: 95.57%
				UAR: 95.59%
				UAP: 95.79%
				Average F1: 95.67%
				MCC: 94.14%
				CK: 93.93%
				GM: 95.58%
			DISFA (39,182 frames)	Acc: 96.06%
				UAR: 96.04%
				UAP: 96.16%
				Average F1: 96.08%
				MCC: 94.78%
				CK: 94.74%
				GM: 96.03%

Acc, Accuracy; AUC, area under curve; CK, Cohen’s kappa; CNN, convolutional neural network; F1, F1-ScoreGM, geometric mean; LSTM, long short-term memory; MAE, mean absolute error; MCC, Matthew’s correlation coefficient; MSE, mean squared error; PR-AUC, precision-recall area under the curve; Pre, precision; Rec, recall; UAP, unweighted average precision; UAR, unweighted average recall.

**Table 5:** Frequency distribution of video frames in the UNBC-McMaster and DISFA databases by PSPI scores and PSPI groups.

PSPI score	UNBC-McMaster database		DISFA database	
	Frequency by PSPI score	Frequency by PSPI group	Frequency by PSPI score	Frequency by PSPI group
0	40,029	2483*	90,309	9025*
1	2909	2909	9973	9973
2	2351	3763	12,194	10,309*
3	1412		8447	
4	802	1697	4127	9875
5	242		1661	
6	270		1526	
7	53		996	
8	79		435	
9	32		503	
10	67		317	
11	76		169	
12	48		108	
13	22		33	
14	1		0	
15	5		0	
Total	48,398	10,852	130,798	39,182

*Random under-sampling of over-represented PSPI groups was performed to create more balanced study datasets with smaller total numbers of video frames for training and testing the pain intensity classification model.

It can be noted from Table 4 that most of the presented models have been developed using the UNBC-McMaster dataset. These are deep learning models since deep learning has high image classification ability and uses deep end-to-end networks. These end-to-end deep learning models have exponential computational complexity. To handle this problem, we proposed a deep feature engineering model that used a shallow classifier (kNN). Other researchers have used ensemble classifiers, Softmax, or deep classifiers. Our model attained better classification performance with the kNN classifier: 1.43% higher classification accuracy than the next best performer in Table 4 (Bargshady^35^). To the best of our knowledge, we are the first team to use the DISFA dataset, for which we report excellent accuracy of 96.06% for detecting different pain intensities.

To calculate the time burden of the proposed shutter blinds-based facial image classification model, asymptotic notation—Big O notation—has been used. We used variables to explain this calculation better. In the blind-based deep feature extraction phase, the raw image and blinds are used as inputs to the deep feature generator. Thus, the time complexity of the feature extraction phase is $O\left( {Sd + tsd} \right)$. Herein, $t$ is the number of blinds, $s$ represents the size of each blind, $d$ is the complexity of the pretrained deep learning model (the deep learning model is utilized as feature generator), and $S$ is the size of the facial image. In the feature selection phase, the INCA method is used, and the time complexity of this method is $O\left( {N + rc} \right)$. The $N$ indicates the computational complexity and $r$, the iteration range of the INCA. The $c$ signifies the computational complexity of the classifier used. The final phase of the model is classification and its time complexity is $O\left( c \right)$. In total, the time complexity of the developed model is $O\left( {Sd + tsd + N + rc + c} \right)$. This result shows that the proposed method has linear computational complexity.

The highlights of the current study are:

- A novel patch-based shutter blinds feature engineering model was proposed.
- Transfer learning-based pre-trained DarkNet19 was used in our model to create deep features due to its superior performance. In fact, the exact transfer learning model need not be final. The shutter blinds base model can be flexibly coupled to other pre-trained networks (e.g., DarkNet53, MobileNetV2, and ResNet50), feature selection functions, and classifiers to evolve new versions for further evaluation.
- We are the first research team to use DISFA to detect pain levels. Furthermore, both datasets used contain over 10,000 images.
- Robust model performance was attained with a shallow fine kNN classifier using tenfold cross-validation.
- The shutter blinds base model (pseudocode given in Algorithm 1) is agnostic and can be applied to solve diverse computer vision problems.
- We trained and tested the shutter blinds-based model on two large facial image datasets and attained excellent classification performance, with overall accuracy rates exceeding 95%.

Limitations of this work are given below:

- A shallow classifier (kNN) with default hyperparameters was used to calculate the results to economize on the computational demand. While our results are excellent, it is possible that even better results could have been obtained by using optimization methods to tune parameters of the kNN or using advanced classifiers such as deep neural network.
- We trained and tested our model on two datasets. More pain image datasets can be used to evaluate the performance of the proposed automated system to enhanced its generalizability.

Our proposed shutter blinds-based pain intensity classification model outperformed other published deep models.

The presented pain intensity classification model comprises (1) novel feature extraction using dynamic-sized horizontal patches or “shutter blinds” and pre-trained DarkNet19 deep network; (2) INCA-based optimal feature vector selection; and (3) classification using standard shallow fine kNN. High pain intensity classification results were obtained using facial images from two public databases of facial expressions videos. Individual video frames have been encoded and scored using validated FACS and PSPI methodology, respectively. The model attained 95.57% and 96.06% accuracy rates on 10,852 and 39,182 facial images derived from the UNBC-McMaster Shoulder Pain Expression Archive and DISFA databases, respectively. As mentioned, our study is the first to use the general facial recognition DISFA database to investigate pain intensity, which yielded similar excellent results for our model compared with the dataset derived from the UNBC-McMaster database. The high performance of our model suggests that it can be implemented in the clinic for non-verbal detection of pain using facial images.

## Methods

The publicly available University of Northern British Columbia (UNBC)-McMaster Shoulder Pain Expression Archive Database^21^ and Denver Intensity of Spontaneous Facial Action (DISFA) Database^42^ were used to train and test the proposed method in this paper. To obtain permission for data usage, a request has been sent together with an explanation for the research. All methods were carried out in accordance with relevant guidelines and regulations. The attributes/properties of the two pain datasets have been tabulated in Table 5.

The details about these two used datasets have been given in below.

*UNBC-McMaster Shoulder Pain Expression Archive Database:* The database comprised 200 videos of the facial expressions of 129 subjects (63 male, 66 female) with self-reported shoulder pain, which had been recorded during left and right shoulder range-of-movement testing. The videos contained a total of 48,398 frames, in each of which individual identifiable facial actions were encoded in terms of 44 possible action units (AUs)^43^ of the validated facial action coding system (FACS)^29^ by certified coders. Among these, facial actions that are potentially related to pain include brow-lowering (AU4), cheek-raising (AU6), eyelid tightening (AU7), nose wrinkling (AU9), upper-lip raising (AU10), and eye-closure (AU43). Except for AU 43, every action was coded on a 5-level intensity scale. Therefore, the validated Prkachin and Solomon Pain Intensity (PSPI) Scale^44^ was calculated for each frame as follows:

1 $$ Pain = AU4 + \left( {AU6\, or\, AU7} \right) + \left( {AU9\, or\, AU10} \right) + AU43 $$

The PSPI score ranged from 0 (“no pain”) to 16 (“strong pain”). The video frames in the UNBC-McMaster database had a maximum score of 15, and the database was unbalanced across PSPI score levels (Table 2). To create a balanced dataset for training and testing the proposed method, video frames coded as “no pain” were randomly under-sampled. All resultant frames regrouped into four discrete classes of increasing pain intensities: PSPI = 0, PSPI = 1, 2 ≤ PSPI ≤ 3, and PSPI > 3, with frequencies (and relative percentages) of 2483 (22.88%), 2909 (26.81%), 3763 (34.68%), and 1697 (15.64%), respectively (Table 2). UNBC-McMaster Shoulder Pain Expression Archive Database is available at https://sites.pitt.edu/~emotion/um-spread.htm.

*DISFA Database:* The database comprised 130,798 video frames of the facial expressions of 27 young adults (15 male, 12 female), which had been recorded with a stereo camera while they were being shown video clips intended to elicit spontaneous emotional expressions^42^. The database was neither designed to study pain intensity nor actual pain stimuli applied during data acquisition. Nevertheless, AUs known to be implicated in the pain response were identifiable in individual video frame recordings of some subjects after they had been shown certain video clips. For training and testing the proposed pain intensity classification model, all video frames were coded using FACS, and the corresponding PSPI scores were calculated. The video frames in the DISFA database had a maximum score of 13, and the database was unbalanced across PSPI score levels (Table 5). The video frames were grouped into four groups—PSPI = 0, PSPI = 1, 2 ≤ PSPI ≤ 3, and PSPI > 3, with frequencies (and relative percentages) of 9025 (23.03%), 9973 (25.45%), 10,309 (26.31%), and 9875 (25.20%), respectively—after random under-sampling of the first and third groups to create a balanced study dataset (Table 5). Denver Intensity of Spontaneous Facial Action (DISFA) Database is available at https://paperswithcode.com/dataset/disfa.

*DarkNet19:* The DarkNet19 is a lightweight convolutional neural network (CNN)^34^ that is widely used in image classification models, e.g., in the You Look Once (YOLO) framework. Moreover, it is the backbone of the YOLOv2. DarkNet19 uses 3 × 3 and 1 × 1 convolutions to extract high-level features and pooling layers for compression. A pre-trained version of the DarkNet19 has been trained on ImageNet1K, an image dataset comprising about 1.3 million images with 1,000 object categories. In the transfer learning mode, global average pooling was used to extract deep features from DarkNet19.

**Figure 4:**
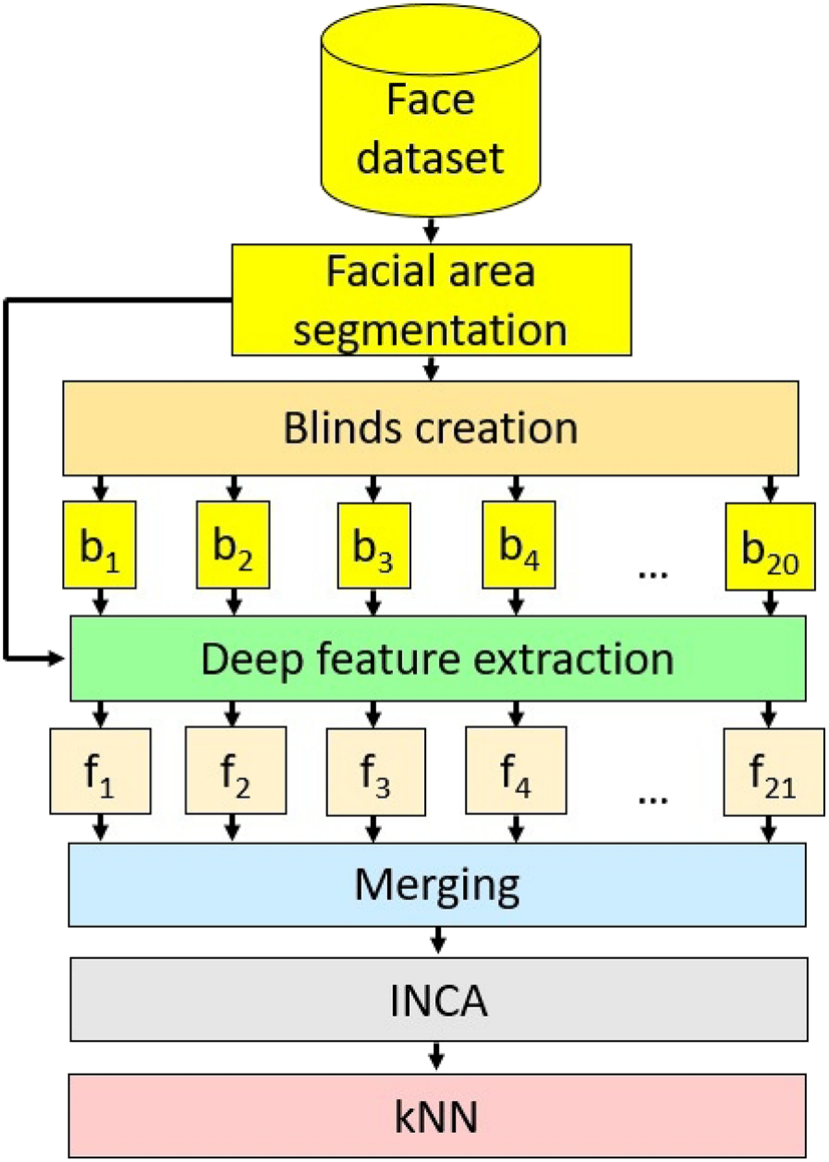
Schema of the proposed model based on shutter blinds-based deep feature extraction.

The challenge of identifying pain using facial images can be posed as a multiclass classification problem. Two distinct study datasets derived from the publicly accessible UNBC-McMaster and DISFA databases of video recordings of facial images, which have been stratified into four labeled PSPI groups using common validated FACS methodology, served as the ground truth. We built a cognitive classification model for pain intensity using novel shutter blinds-based deep feature extraction coupled with iterative neighborhood component analysis (INCA) feature selection (Fig. 4). First, the face portion of each video frame was segmented. It was resized to a 224 × 224 image, which was then divided into 21 dynamic-sized horizontal patches, each of which could be half, quarter, one-seventh, or one-eighth of the resized segmented facial image (Fig. 4). Next, pre-trained DarkNet19 was used to extract 1000 deep features from each of the 21 horizontal patches as well as the original facial image. These features were concatenated to generate a final feature vector of length 22,000. INCA was deployed to choose the most discriminative features, which constituted the best feature vectors of input images derived from the UNBC-McMaster and DISFA datasets with vector lengths of 768 and 834, respectively. With INCA, the optimum vector lengths with the most discriminative features for the different datasets were calculated with a loss function using kNN with tenfold cross-validation. According to the calculated loss values, 768 and 834 among 22,000 extracted features were selected as the most discriminative features for the UNBC-McMaster and DISFA datasets, respectively. The selected features were fed to a fine k-nearest neighbor (kNN) classifier^45^ for 4-class classification.

The pseudocode of the proposed model is shown in Algorithm 1.

**Algorithm 1**. Pseudocode of proposed shutter blinds-based model for classification of pain levels.


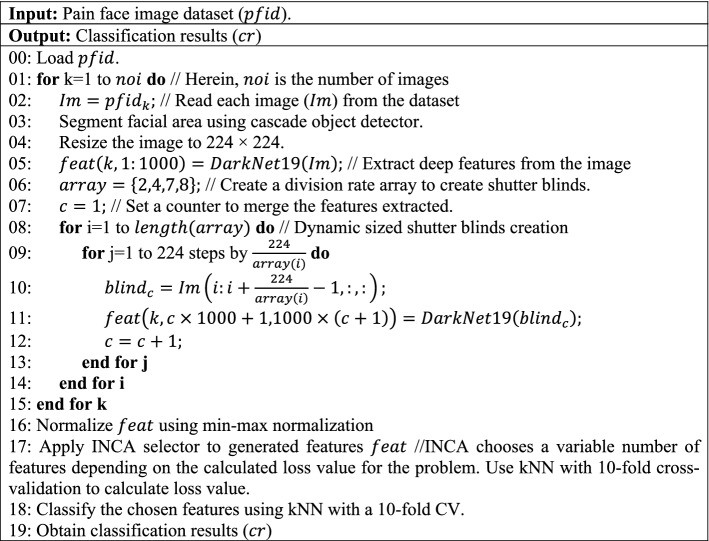


Novel feature engineering methodology is the most important and inventive contribution to this study. In the preprocessing phase, facial area segmentation and resizing of the segmented facial image into standardized 224 × 224 dimensions were performed. Each resized segmented facial image was divided into 21 dynamic-sized horizontal patches—“shutter blinds”—comprising two 112 × 224, four 56 × 224, seven 32 × 224, and eight 28 × 224 patches. DarkNet19^34^, a convolutional neural network pre-trained on the 1,000-category ImageNet image dataset, was used to extract 1000 features from each patch and the undivided resized segmented face image. Transfer learning is computationally efficient and has yielded high classification performance in computer vision applications^46^. Finally, the extracted features were concatenated to form a final feature vector of length 22,000.

Our method used patch-based deep feature extraction to extract only the most valuable features from the facial area (see Line 03 of Algorithm 1). In our novel patch division model, 21 patches were created for downstream feature extraction, in addition to the original raw image. As such, both local and global features could be extracted from 22 (= 21 patches + 1 raw facial image) image inputs. We utilized a pre-trained CN, DarkNet19, as the deep feature extractor to generate feature 22 vectors. These feature vectors were merged into a final feature vector of length 22,000 (= 22 × 1000). The shutter patch-based feature extraction process is defined in Lines 04–15 of Algorithm 1. In the feature selection phase (see lines 15–16 of Algorithm 1), each feature was normalized using min–max normalization, and INCA feature selector was then applied. INCA is an iterative version of neighborhood component analysis. First, neighborhood component analysis was applied to the extracted features to generated qualified indexes. Using these qualified indexes, a loop, and a loss value calculator, classification performances were calculated, and the feature vector with the best classification performance was selected. The optimal number of features were selected using the INCA selection method. In the classification phase (see Line 18 of Algorithm 1), a kNN classifier with tenfold cross-validation was used.

The steps of the shutter blinds-based deep feature extraction have been pseudo-coded in lines 01 to 15 of Algorithm 1 and are also explained below:

***Step 1:*** Divide the image into 21 dynamic-sized horizontal patches—“shutter blinds”—comprising two 112 × 224, four 56 × 224, seven 32 × 224, and eight 28 × 224 patches.

***Step 2:*** Extract deep features from the generated shutter blinds and undivided raw face image by applying pre-trained DarkNet19. In this work, we used pre-trained DarkNet19 with default settings to generate features without fine-tuning or optimization operations.

***Step 3:*** Merge the extracted deep features to obtain the final feature vector.

INCA^47^ is an iterative and improved version of neighborhood component analysis^48^, which is itself the feature selection counterpart of kNN. INCA efficiently selects the most discriminative features and the optimal number of features by trial-and-error using two essential parameters: range of iteration and loss function, which have been set at [100, 1000], respectively, in this work. Deep networks lead to high computational costs. Therefore, the main purpose of defining an iteration is to decrease computational cost. Moreover, the misclassification rates of the various selected feature vectors of different lengths were calculated and compared to choose the optimal feature vectors, which could be of different lengths depending on the image input from the different datasets.

***Step 4:*** Apply the INCA function with given parameters to the generated 22,000 features to select 768 and 834 features from input facial images of the UNBC-McMaster and DISFA datasets, respectively.

We used a simple distance-based classifier without an additional optimization algorithm in this work. From testing the 30 standard shallow classifiers in the MATLAB (2021a) classification learner toolbox, fine kNN^45^ was found to deliver the best-calculated results for classifying the selected features in our novel feature extraction model and was thus chosen to be included in our final model.

***Step 5:*** Feed the selected features to the fine kNN classifier for automated classification using ten-fold cross-validation.

### Future directions

We have presented a new deep feature engineering model named shutter blinds. Our proposed shutter blinds method uses horizontal patches to extract features of the whole face and local facial areas. The model’s classification performance for detection of pain intensities was tested on two publicly available facial image datasets. We plan to develop more accurate and generalizable pain intensity classification models by training and testing them on larger facial image datasets collected from more subjects of diverse ethnicities and annotated using validated FACS and PSPI methodology. Such a model can assist doctors in detecting and managing pain in patients proactively. Our future works include:

- Based on our and other researcher’s success with patch-based deep feature extraction models, we shall continue to develop new patch-based deep feature engineering computer vision models.
- Our proposed computer vision model is a parametric image classification method. In this method, DarkNet19 was used as a deep feature extractor, INCA was used to choose the most informative features, and kNN used for classification. It is possible to combine other deep feature extractors, feature selectors, classifiers, and variable shutter blinds-based image classification methods. On our priority list is a proposal to build an ensemble ResNet-based shutter blind architecture.
- The proposed non-fixed size horizontal patch division model can be combined with transformers to create a new generation and potentially more accurate, transformers-based computer vision networks that can detect pain intensities.
- This work used the proposed shutter blinds on human faces to detect pain levels. It is conceivable that similar models can be applied to classify pain levels in animals. As such, customized models can be used in medical as well as veterinary centers.

## Data Availability

The public data presented in this study are available from UNBC-McMaster Shoulder Pain Expression Archive Database (https://sites.pitt.edu/~emotion/um-spread.htm) and Denver Intensity of Spontaneous Facial Action (DISFA) Database (https://paperswithcode.com/dataset/disfa).
